# Evidence-based practice a relevant piece to update knowledge in mental health nursing

**DOI:** 10.1192/j.eurpsy.2021.1592

**Published:** 2021-08-13

**Authors:** C. Laranjeira, A. Querido, O. Valentim

**Affiliations:** 1 Citechcare, Polytechnic of Leiria, Leiria, Portugal; 2 School Of Health Sciences, Polytechnic of Leiria, Leiria, Portugal

**Keywords:** Evidence-based practice, Nursing education, Mental Health Nursing, Journal Club

## Abstract

**Introduction:**

Web Journal Club as a collaborative learning method, is an effective method to enhance the knowledge base of nursing students, their presentation skills, problem-solving skills and ability to critically appraise literature.

**Objectives:**

To describe the experience of a online journal club on education of MHN undergraduate students.

**Methods:**

We implemented a journal club in the online classroom with a total of 24 portuguese undergraduate students enrolled in clinical training of MHN (sixth semester). Over a two-week period, five 2-hour online journal club sessions were conducted in April 2020. During each session, five journal articles were presented synchronously to a live online audience via the Zoom Classroom technology. After all sessions, students were invited by e-mail to complete an anonymous and voluntary online questionnaire via Google Forms.

**Results:**

All students were all very positive about the journal club sessions and found the opportunity to discuss and reflect on practice issues in depth very helpful. They found the sessions supportive, they helped to bond the group, they learnt a great deal from each others experiences, and they felt that they gained in confidence as a group. Survey results also indicated that few participants experienced technical difficulties during sessions.

**Conclusions:**

This pedagogical practice enhances gains in the various actors involved: 1) in students, contributing to their learning process and acquisition of competences, articulating research and clinical practice; and 2) lastly, even more indirectly, in people receiving care, since a evidence-based practice ensures safe and quality of nursing care delivery.

